# Myocardial blood flow assessment with ^82^rubidium-PET imaging in patients with left bundle branch block

**DOI:** 10.6061/clinics/2015(11)02

**Published:** 2015-11

**Authors:** Andréa Falcão, William Chalela, Maria Clementina Giorgi, Rodrigo Imada, José Soares, Renata Do Val, Marco Antonio Oliveira, Marisa Izaki, Roberto Kalil Filho, José C Meneghetti

**Affiliations:** Instituto do Coração (InCor) do Hospital das Clínicas da Faculdade de Medicina da Universidade de São Paulo, Serviço de Medicina Nuclear e Imagem Molecular, São Paulo/SP, Brasil.

**Keywords:** Myocardial Blood Flow, Left Bundle Branch Block, Positron Emission Tomography (PET), Myocardial Perfusion Imaging, Coronary Flow Reserve

## Abstract

**OBJECTIVES:**

Perfusion abnormalities are frequently seen in Single Photon Emission Computed Tomography (SPECT) when a left bundle branch block is present. A few studies have shown decreased coronary flow reserve in the left anterior descending territory, regardless of the presence of coronary artery disease.

**OBJECTIVE:**

We sought to investigate rubidium-82 (^82^Rb) positron emission tomography imaging in the assessment of myocardial blood flow and coronary flow reserve in patients with left bundle branch block.

**METHODS:**

Thirty-eight patients with left bundle branch block (GI), median age 63.5 years, 22 (58%) female, 12 with coronary artery disease (≥70%; GI-A) and 26 with no evidence of significant coronary artery disease (GI-B), underwent rest-dipyridamole stress ^82^Rb-positron emission tomography with absolute quantitative flow measurements using Cedars-Sinai software (mL/min/g). The relative myocardial perfusion and left ventricular ejection fraction were assessed in 17 segments. These parameters were compared with those obtained from 30 patients with normal ^82^Rb-positron emission tomography studies and without left bundle branch block (GII).

**RESULTS:**

Stress myocardial blood flow and coronary flow reserve were significantly lower in GI than in GII (*p*<0.05). The comparison of coronary flow reserve between GI-A and GI-B showed that it was different from the global coronary flow reserve (*p*<0.05) and the stress flow was significantly lower in the anterior than in the septal wall for both groups. Perfusion abnormalities were more prevalent in GI-A (*p*=0.06) and the left ventricular ejection fraction was not different between GI-A and GI-B, whereas it was lower in GI than in GII (*p*<0.001).

**CONCLUSION:**

The data confirm that patients with left bundle branch block had decreased myocardial blood flow and coronary flow reserve and coronary flow reserve assessed by ^82^Rb-positron emission tomography imaging may be useful in identifying coronary artery disease in patients with left bundle branch block.

## INTRODUCTION

Noninvasive diagnosis of coronary artery disease (CAD) in left bundle branch block (LBBB) patients is a clinical and methodological dilemma because septal perfusion abnormalities have been demonstrated by myocardial perfusion scintigraphy (gated-SPECT) even in the absence of CAD 1-8. Some evidence has been published that indicates that there is decreased coronary flow reserve (CFR) in the left anterior descending (LAD) territory, regardless of the presence of CAD. However, few studies have investigated the clinical significance of this finding.

Rubidium-82 (^82^Rb) positron emission tomography (PET) stress myocardial perfusion has emerged in the clinical setting as a noninvasive imaging method for diagnosis and risk-stratification that has several advantages compared to Single Photon Emission Computed Tomography (SPECT). In addition to assessing myocardial perfusion, wall motion and left ventricular function, ^82^Rb-PET can quantify global and regional myocardial blood flow (MBF) during both rest and stress, and can also measure CFR, which is important for the clinical management of patients with CAD 9,10.

Hirzel et al. 3 evaluated thallium-201 uptake and made regional MBF measurements using radioactive microspheres in dogs with pacing-induced LBBB. The septal thallium-201 uptake and myocardial flow were significantly decreased compared to that in the lateral wall, which showed that the coronary flow to the septum is limited due to the deranged contraction, i.e., the concept of functional ischemia.

In another invasive study using cardiac catheterization, which evaluated CFR with adenosine in 13 patients with LBBB and normal coronary arteries, Skalidis et al. 11 found a longer time to maximum peak diastolic flow velocity than that in controls. Furthermore, the CFR was significantly lower in the LAD territory than in the right coronary artery (RCA). The reduced CFR was associated with the presence of scintigraphic perfusion abnormalities.

Coronary flow is dependent on both the epicardial vessel and microvascular flow properties 12 and therefore, it is unclear whether LBBB is associated with reduced CFR. Thus, we aimed to evaluate the influence of LBBB on MBF, dipyridamole stress MBF and CFR, as measured with ^82^Rb-PET.

## MATERIALS AND METHODS

The study was performed at the Department of Nuclear Medicine and Molecular Imaging of the Heart Institute of the University of São Paulo Medical School and was approved by the institutional ethics review board.

### Study Design

From February to October 2013, 665 consecutive patients, who were referred for dipyridamole-stress gated-SPECT for the evaluation of known or suspected CAD, underwent ^82^Rb-PET. Of the 51 patients with ECG evidence of LBBB who were defined according to standard criteria (QRS ≥120 ms), 38 were selected (Group I - GI) who met the inclusion criteria: LBBB and sinus rhythm. The median age was 63.5 years, range 58-68 years and 22 were women (58%). Patients with unstable angina, recent myocardial infarction, pacemaker rhythm and atrial fibrillation or flutter were excluded from GI. In addition, we selected 30 patients without LBBB (Group II - GII), who were free from known CAD and had normal ^82^Rb-PET studies (including perfusion, function and blood flow measurements), and matched them based on the same clinical risk factors that are associated with reduced absolute quantitative flow measurements 13,14. The median age was 59.5 years (range 53-65 years) and 17 were women (57%).

### Assessment of CAD

In a subset of patients, a request to perform a coronary anatomy assessment was made by the patient's doctor. Therefore, a cardiac catheterization or coronary computed tomography angiography (CTA) 15 was performed in 31 patients with LBBB. A significant coronary stenosis was defined as ≥70% luminal narrowing in one or more vessels (GI-A, n=12). Based on a finding of no significant stenosis and a normal ^82^Rb-PET perfusion study, patients were categorized into a subgroup with LBBB and no CAD (GI-B, n=26).

### Myocardial Perfusion 82Rb-PET Imaging Protocol

The patients were instructed to fast for 4 h, abstain from caffeine and cigarettes for 24 h and theophyllines for 36 h, and discontinue beta-blocker or calcium-channel blocker medications for 3 days and long-acting nitrates for 6 hours before the study.

Cardiac PET was performed using a Gemini-TOF 64-slice system (Philips Medical Systems, Cleveland, Ohio, USA). Resting images were acquired using a 3-D list mode acquisition over 8 min, after a square wave intravenous injection of ^82^Rb (10 MBq/kg; Jubilant DraxImage) over a 60-second interval.

Dipyridamole was infused intravenously (0.56 mg/kg) over four minutes and the same ^82^Rb activity was administered four minutes later. Stress images were acquired and processed for the resting scan. The symptoms were treated with intravenous aminophylline after the end of image acquisition.

Before rest and after stress, ^82^Rb-PET image acquisitions with two low-dose CT-based attenuation corrections for PET transmission scans were performed (120 kV; 115 mAs; and 0.435 pitch), after a normal end-expiration. The estimated effective radiation dose from the complete PET study (rest/dipyridamole ^82^Rb and 2 CT attenuation corrections) was 3.0 mSv 16.

### Image Processing

Fused CT and emission images were visually evaluated for alignment by an experienced technologist and, if necessary, corrected by manual 3-D translation. The images were reconstructed using a 3-dimensional row-action maximum likelihood algorithm (3-D-RAMLA), with 3 iterations/33 subsets and a medium filter. The entire 8 min of emission data was binned to form a dynamic image sequence (9 x 10 s, 3×30 s, 1×60 s and 2×120 s) for MBF quantification and the last 6 min to form myocardial uptake and ECG-gated images. The images were semi-automatically reoriented to generate short-axis and vertical long-axis slices. The left ventricle ejection fraction (LVEF) was calculated from the rest and stress images using 4D QGS 17 software, version 2012.2.

### Quantitative MBF Measurements

The studies were processed in batch mode using QPET (Cedars-Sinai, Los Angeles, California) to quantify MBF and CFR. In brief, left ventricle (LV) contours were positioned automatically with an algorithm that determines the LV contours from the summed dynamic images data. The 3-D cylindrical region (1 cm diameter, 2 cm length) for the LV input function was automatically placed in the middle of the valve plane and oriented along the long axis of the heart. Dynamic myocardial samples were obtained from the polar map by analyzing all of the time frames within the fixed LV contour boundaries 18.

A standard 1-tissue compartment model was used to quantify MBF, which includes regional uptake and clearance parameters (*K_1_* [mL/min/g of myocardial tissue] and *k_2_* [min^−1^]), blood-to-myocardial spillover fraction and myocardial partial-volume corrections. A previously calibrated ^82^Rb extraction fraction was used to estimate MBF from *K_1_* 19.

Stress and rest MBF were computed for each sample in the polar map, and CFR was calculated as the ratio of stress/rest MBF. Four basal slices were not used for flow analysis due to low counts in the membranous septum. MBF in each vascular territory was then obtained by averaging the polar map segments in the regions of the LAD, RCA, and left circumflex artery (LCX), according to the standard 17 segments of the American Heart Association model 20 and corresponding to the anterior, septal, lateral, inferior, and apical walls.

### Image Interpretation

Rest and stress uptake images were visually analyzed using a 5-point score (0-normal; 1-mild uptake; 2-moderate; 3-severe and 4-no uptake) for relative myocardial perfusion in 17 segments 20. We compared the GI-A and GI-B patients considering the anterior and septal walls as segments 1, 2, 7, 8, 13, and 14 and the lateral and inferior walls as segments 3, 4, 5, 6, 9, 10, 11, 12, 15, and 16. Segment 17 was excluded in this analysis. Two experienced nuclear physicians who were blinded to the patient data analyzed the perfusion images. Summed stress scores (SSS), summed rest scores (SRS) and summed difference scores (SDS) were determined. An SSS ≥4 and/or SDS ≥2 were considered perfusion abnormalities. A rest or stress LVEF <45% was considered to be abnormal.

### Statistical Analysis

Quantitative variables were expressed in medians (25%-75% quartile), while qualitative variables were expressed in percentages. Differences in the quantitative variables between GI and GII were verified by *t* or Mann-Whitney tests conditioned by the normality distribution assumption, which was checked using the Anderson-Darling test and the assumption of homogeneity of variances, which was established by the Levene test. In turn, discrepancies among GI-A, GI-B and GII were verified by ANOVA or Kruskal-Wallis tests, supported by the assumptions of normality and homogeneity of variances. Next, multiple comparisons relative to GI-A *vs*. GI-B and GI-B *vs*. GII were performed using the parametric or nonparametric Dunnett test 21.

Comparisons of the anterior or septal wall with the other walls were evaluated using the paired nonparametric Dunnett test 22. Finally, differences in the qualitative variables were analyzed using the Fisher test. All of the discrepancies were classified as statistically significant considering a significance level at 5%. The calculations were performed using the *R* package, version 3.1.1. 23.

### Ethics

All of the procedures that were performed in studies that involved human participants were in accordance with the ethical standards of the institutional and national research committee and with the 1964 Helsinki declaration and its later amendments or comparable ethical standards. This article does not contain any studies with animals performed by any of the authors. Informed consent was obtained from all individual participants included in the study.

## RESULTS

### Clinical and demographic characteristics

The patient demographics are summarized in Table 1. In GI patients, 12 had CAD (32%, GI-A) with 2- or 3-vessel disease in 67%, 1-vessel disease in 25% and a myocardial bridge in 8%. Twenty-six patients (68%, GI-B) had normal coronary arteries or <70% stenosis and normal ^82^Rb-PET perfusion. The median ages were not significantly different (GI *vs*. GII, *p*=1.00; GI-A *vs*. GI-B *vs*. GII, *p*=0.06). The female gender was predominant in all groups. Furthermore, the main clinical risk factors, such as hypertension, dyslipidemia and diabetes, were also not significantly different between the groups.

### Quantitative MBF Measurements

Global and regional values in GI and GII are summarized in Table 2. The stress flow and CFR were significantly lower in GI than in GII in all of the walls (*p*<0.001), whereas the rest flow was lower only in the septal and apical walls (*p*=0.02 and 0.01, respectively). A comparison of the anterior or septal walls with the other walls in GI revealed that the rest flow, stress flow and CFR were significantly lower in the anterior wall and less so in the septal wall. However, the rest and stress flows were also significantly lower in the septal than in the inferior wall (Table 2). A comparison between GI-A and GI-B did not show any significant flow differences, except for the global CFR (*p*=0.05) and could identify CAD in LBBB (Table 3). Moreover, GI-A and GI-B had significantly lower stress flow in the anterior wall than in the septal wall. Thus, flow in the anterior wall was more affected by LBBB than that in the septal wall, independent of the presence of CAD. CFR in the apical and lateral walls were lower in GI-B than in GII, which could explain a pattern characteristic of LBBB in the absence of CAD.

### Myocardial Perfusion 82Rb-PET Imaging and LV function

**Figure 1:**
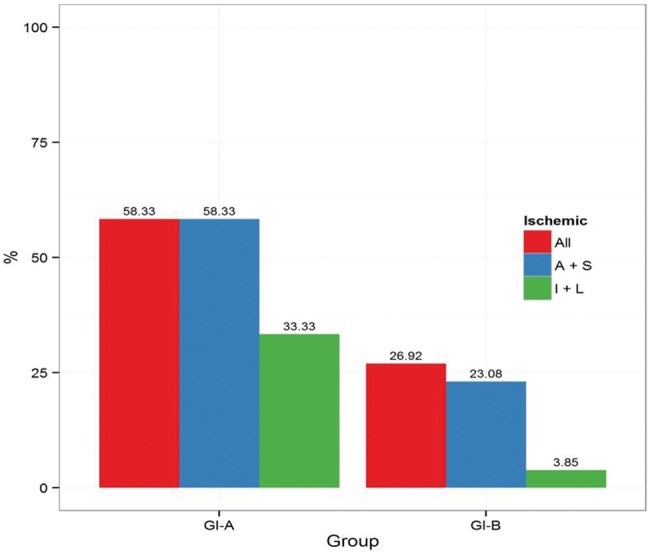
Myocardial perfusion abnormalities were more prevalent in GI-A than in GI-B (*p*=0.03), and ^82^Rb-PET myocardial perfusion could discriminate between the groups in all LV territories. All of the values are expressed in terms of the number of patients. A = anterior; S = septal; I = inferior; L = lateral.

Twenty-four patients in GI (63%) had no significant perfusion abnormalities and all patients in GII had normal perfusion. Moreover, the perfusion abnormalities (SSS ≥4 and/or SDS ≥2) tended to be more prevalent in the presence of CAD (GI-A *vs*. GI-B; *p*=0.06). We found statistically significant differences between the groups when we considered perfusion abnormalities in the anterior and/or septal walls (*p*=0.04) as well as in the inferior and/or lateral walls (*p*=0.003) (Figure 1).

LVEF values and LV cavity volumes are summarized in Tables 4 and 5. Twenty-five patients in GI (66%) had LV dysfunction at rest and all of the GII subjects had normal LVEF. There were significantly lower rest and stress LVEFs in GI than in GII (*p*<0.001), but no significant differences between GI-A and GI-B (*p*=ns). However, GI-A had a high prevalence of LVEF dysfunction (<45%) only during stress (*p*=0.02).

## DISCUSSION

An evolution in cardiovascular imaging with PET-CT systems being incorporated into the clinical setting has been occurring since the early 2000s. This trend is partially driven by the fact that cardiac PET-CT offers a noninvasive method to assess relative myocardial perfusion, LV function, MBF and calcium score (15). The importance and independent prognostic values of MBF and CFR using ^82^Rb-PET, beyond relative myocardial perfusion image interpretation, has already been established. Worse patient outcomes and a higher incidence of cardiac events (death and myocardial infarction) have been demonstrated in cases with global CFR <2 mL/min/g in patients with normal and abnormal relative myocardial perfusion 24,25.

Despite the low prevalence of LBBB in the general population 26, several studies have reported its frequent association with heart disease. In the Framingham study, CAD was found in 40% of LBBB patients and was associated with a fourfold increase in the risk of cardiovascular mortality 27. Dynamic alterations in the cardiac cycle produced by LBBB are known: asynchrony of contraction in the ventricles; reduction of LV diastolic time; abnormal septal motility; and abnormal septal ejection fraction 3,4,28,29. Such alterations confound the noninvasive diagnosis of CAD, especially with the exercise gated-SPECT test 1,4-8,. The mechanisms that are responsible for abnormal findings may be associated with functional ischemia 4,5,28 or abnormal CFR 3. However, few studies have reported patterns in MBF and CFR in LBBB, and they did not include patients with CAD. A study by Masci et al. 33 evaluated a small group of patients with dilated cardiomyopathy and LBBB with 2-[18F]fluoro-2-deoxyglucose and 13N-ammonia-PET. They did not demonstrate significant differences in MBF and CFR between patients with and without LBBB. However, the myocardial glucose metabolic rate was lowest in the septum of LBBB patients. In another study 34, 10 LBBB patients were analyzed with ^15^O-water PET and the study results showed that the septal/lateral MBF ratio was 19% lower than that in the controls, as a result of functional alterations. Our study assessed LBBB patients and showed that stress MBF as well as CFR were significantly lower in all of the walls in a larger LBBB population compared to the controls. We were careful to compare 2 homogeneous populations that were matched for the presence of primary clinical risk factors, which are associated with reduced stress flow and CFR 13, because coronary atherosclerosis is common in middle-aged people. Our results confirm previous findings of reduced rest flow in the anterior and septal walls, which demonstrates that LBBB affects MBF and CFR, probably because of abnormal LV activation and uncoordinated contractions 29. Another confirmatory finding was that globally and for most of the walls, CFR was abnormal in the presence of CAD (<2 mL/min/g) (24), which demonstrates there is an incremental influence of the presence of CAD on LBBB and CFR measurement by ^82^Rb-PET that could aid in the identification of CAD and risk-stratification of LBBB patients.

Our results suggest that resting MBF in the inferior wall may not be influenced by the presence of LBBB (in GI-A patients, stress MBF was lower in the anterior than in the septal wall and even lower in the inferior wall). In GI-B patients, stress and rest flow were also lower in the anterior than in the septal and inferior walls. It appears that LBBB did not interfere with the evaluation of MBF and CFR in the inferior wall or the abnormal activation of the LV because LBBB is more proximal (anterior and high septum areas), sparing the inferior or even lateral walls, which are opposite and more distal. In the presence of LBBB (both with and without CAD), stress MBF was also lower in the anterior than in the septal wall. The septal wall showed a smaller MBF impairment than that in the anterior wall, which could be explained by the fact that the septum is a less extensive area compared to others and thus, MBF may have a lower expression or be underestimated in a quantitative evaluation.

Our study also suggests that ^82^Rb-PET relative myocardial perfusion could discriminate between LBBB patients with and without CAD, even when considering the LAD territory alone. Perfusion abnormalities were more prevalent in the presence of CAD. Although anterior and septal abnormalities have been described in most similar studies in the absence of CAD, perfusion abnormalities may also result from technical imaging issues (e.g., partial volume effect and attenuation artifacts - mainly with SPECT), functional ischemia, CFR alterations caused by the LV dynamic asynchrony, or because of subclinical myocardial disease 4,5,28.

Rest and stress LVEFs in LBBB patients were also lower than those in GII. As expected, GI-B had less LV dysfunction than GI-A at stress and it was associated with CAD. Furthermore, GI-A on average had a lower LVEF response to stress compared to GI-B. Nevertheless, even in the absence of established CAD, patients with LBBB demonstrated a decreased LV reserve and might not respond appropriately to stress 35.

The mechanisms that underlie the reduced MBF and CFR in nonischemic cardiomyopathy can include endothelial dysfunction, macro- and microvascular obstruction, vascular remodeling and extravascular compressive forces 36,37, which could have contributed to reduced MBF, CFR and even LV dysfunction in GI-B. A recent study demonstrated that impairment in CFR is common in both ischemic and nonischemic cardiomyopathy. CFR ≤1.65 mL/min/g by PET imaging in this population was associated with higher major adverse cardiovascular events 38.

Thus, the presence of CAD had a profound effect on CFR and also on traditional relative myocardial perfusion and LV function measures, with a higher prevalence of perfusion and LVEF abnormalities in GI-A. There was a detrimental influence of LBBB over ventricular dynamics, independent of the presence of CAD. These functional abnormalities may be associated with abnormal MBF, perfusion, and function. According to our data, an advantage of using ^82^Rb-PET is that it could provide additional information for the assessment of CAD in LBBB patients.

### Study limitations

Our study has some limitations. Despite the fact that ^82^Rb-PET has several advantages compared with SPECT studies, one disadvantage is the inability to perform ^82^Rb-PET in association with exercise stress tests, due to the short half-life of the tracer. However, in LBBB, pharmacological stress is preferable to exercise perfusion imaging for both diagnosis and risk stratification 20.

This was a single-center observational study that was performed with a select population of LBBB patients. Thus, its results may not be applicable to all such patients because the great diversity of clinical situations in which LBBB can be present may yield different results. Moreover, cardiac catheterization or CTA were not available in all LBBB patients. Usually, the absence of relative perfusion defects in PET-CT studies is associated with non-significant CAD 39. Finally, the high prevalence of LV dysfunction in the absence of CAD might be related to cardiomyopathy, but alterations in MBF and CFR on ^82^Rb-PET in these patients were expected even in the absence of CAD 38.

In the present study, we showed that LBBB patients who were assessed by ^82^Rb-PET have decreased MBF and CFR in all LV territories. Moreover, these measures are important diagnostic tools for patients with LBBB and suspected CAD, which might be identified with a CFR evaluation.

## Figures and Tables

**Table 1:** Comparison of demographic characteristics among groups.

	GI (n=38)	GI-A (n=12)	GI-B (n=26)	GII (n=30)	*p*-value
**Age (yrs)**	63.5 (58.25 - 68)	65 (62 - 69.25)	63.5 (58 - 67.75)	59.5 (53 - 64.75)	0.06
**BMI (kg/m^2^)**	25.3 (23.45 - 28.7)	25.9 (24.75 - 28.58)	24.8 (22.8 - 28.7)	27.85 (25.12 - 29.37)	0.23
**Female**	58%	67%	54%	57%	0.76
**FH of CAD**	30%	33%	29%	8%	0.13
**Hypertension**	73%	83%	67%	73%	0.64
**Diabetes**	39%	58%	29%	29%	0.20
**Dyslipidemia**	61%	58%	62%	52%	0.83
**Smoking**	30%	50%	19%	16%	0.07
**Prior MI**	22%	58%	0%	0%	< 0.001
**Prior PCI**	3%	8%	0%	0%	0.21
**Prior CABG**	6%	17%	0%	0%	0.04

CAD = coronary artery disease; BMI = body mass index; FH of CAD = family history of CAD; MI = myocardial infarction; PCI = percutaneous coronary intervention; CABG = coronary artery bypass graft surgery.

**Table 2:** Results of global and regional ^82^Rb-PET myocardial blood flow measurements during stress and rest and CFR in GI and GII.

	GI (n=38)	GII (n=30)	*p-*value
**Stress (mL/min/g)**			
** ANT**	1.38 (1.07 - 1.99)	2.33 (1.74 - 3.01)	< 0.001
** SEP**	1.75 (1.31 - 2.33) Δ	2.95 (2.23 - 3.39)	< 0.001
** APX**	1.25 (0.96 - 1.90)	2.31 (1.87 - 2.75)	< 0.001
** LAT**	1.68 (1.43 - 2.36) Δ	2.52 (2.03 - 3.04)	< 0.001
** INF**	1.99 (1.48 - 2.95) †Δ	2.72 (2.17 - 3.49)	0.001
** GLOBAL**	1.64 (1.22 - 2.54)	2.54 (1.99 - 3.15)	< 0.001
**Rest (mL/min/g)**			
** ANT**	0.66 (0.54 - 0.94)	0.84 (0.64 - 0.98)	0.07
** SEP**	0.75 (0.62 - 1.04) Δ	0.94 (0.75 - 1.15)	0.02
** APX**	0.65 (0.57 - 0.95)	0.86 (0.66 - 1.08)	0.01
** LAT**	0.79 (0.62 - 0.97) Δ	0.86 (0.72 - 1.03)	0.18
** INF**	0.91 (0.68 - 1.13) †Δ	0.88 (0.70 - 1.21)	0.44
** GLOBAL**	0.74 (0.62 - 1.07)	0.86 (0.73 - 1.10)	0.11
**Coronary Flow Reserve**			
** ANT**	2.15 (1.74 - 2.76)	2.75 (2.36 - 3.55)	0.003
** SEP**	2.48 (1.82 - 3.11) Δ	3.12 (2.62 - 3.46)	0.008
** APX**	1.94 (1.31 - 2.81)	2.74 (2.20 - 3.31)	0.001
** LAT**	2.30 (1.82 - 2.64)	2.96 (2.53 - 3.67)	< 0.001
** INF**	2.44 (1.95 - 3.09)	3.17 (2.50 - 3.95)	0.005
** GLOBAL**	2.46 (1.86 - 2.82)	3.03 (2.46 - 3.72)	0.007

ANT = anterior wall; SEP = septal wall; APX = apical wall; LAT = lateral wall; INF = inferior wall.

† *p*<0.05 in a comparison between the septal and other walls in GI;

Δ *p*<0.05 in a comparison between the anterior and other walls in GI.

**Table 3:** Results of the comparison of global and regional ^82^Rb-PET myocardial blood flow among groups.

Wall	GI-A (n=12)	GI-B (n=26)	*p-*value♦	GII (n=30)	*p-*value*
**Stress (mL/min/g)**					
** ANT**	1.16 (0.82 - 1.89)	1.48 (1.20 - 1.99)	0.53	2.33 (1.74 - 3.01)	< 0.01
** SEP**	1.48 (1.15 - 2.29) Δ	1.88 (1.53 - 2.33) Δ	0.73	2.95 (2.23 - 3.39)	< 0.01
** APX**	1.12 (0.76 - 1.73)	1.29 (1.09 - 1.90)	0.67	2.31 (1.87 - 2.75)	< 0.01
** LAT**	1.70 (1.24 - 2.10)	1.65 (1.44 - 2.36)	0.96	2.52 (2.03 - 3.04)	< 0.01
** INF**	1.92 (1.48 - 2.33) Δ	2.08 (1.51 - 2.95) †Δ	0.73	2.72 (2.17 - 3.49)	0.03
** GLOBAL**	1.56 (1.13 - 2.05)	1.75 (1.44 - 2.54)	0.51	2.54 (1.99 - 3.15)	0.01
**Rest (mL/min/g)**					
** ANT**	0.72 (0.55 - 1.02)	0.66 (0.54 - 0.90)	0.28	0.84 (0.64 - 0.98)	0.28
** SEP**	0.74 (0.62 - 1.12)	0.75 (0.63 - 0.92) Δ	0.14	0.94 (0.75 - 1.15)	0.14
** APX**	0.72 (0.60 - 1.07)	0.64 (0.56 - 0.86)	0.45	0.86 (0.66 - 1.08)	0.02
** LAT**	0.86 (0.72 - 1.23)	0.68 (0.60 - 0.96)	0.10	0.86 (0.72 - 1.03)	0.10
** INF**	0.94 (0.77 - 1.27) †Δ	0.79 (0.64 - 1.05) Δ	0.50	0.88 (0.70 - 1.21)	0.50
** GLOBAL**	0.80 (0.66 - 1.11)	0.72 (0.62 - 1.04)	0.33	0.86 (0.73 - 1.10)	0.33
**Coronary Flow Reserve**					
** ANT**	1.86 (1.18 - 2.49)	2.31 (1.98 - 2.76)	0.31	2.75 (2.36 - 3.55)	0.08
** SEP**	1.92 (1.31 - 2.64)	2.62 (2.13 - 3.16) Δ	0.16	3.12 (2.62 - 3.46)	0.21
** APX**	1.40 (1.14 - 1.98)	2.00 (1.57 - 2.96)	0.25	2.74 (2.20 - 3.31)	0.04
** LAT**	2.04 (1.10 - 2.41)	2.44 (1.90 - 2.66)	0.36	2.96 (2.53 - 3.67)	0.01
** INF**	2.05 (1.40 - 2.49)	2.77 (2.31 - 3.13)	0.09	3.17 (2.50 - 3.95)	0.20
** GLOBAL**	1.94 (1.20 - 2.58)	2.59 (2.16 - 2.95)	0.05	3.03 (2.46 - 3.72)	0.28

CAD = coronary artery disease; ANT = anterior wall; SEP = septal wall; APX = apical wall; LAT = lateral wall; INF = inferior wall.

♦ Comparison between GI-A and GI-B; * Comparison between GII and GI-B;

† refers to the comparison between the septal and other walls in GI-B;

Δ refers to the comparison between the anterior and other walls in GI-A or in GI-B.

**Table 4:** Results of the comparison of left ventricle ejection fraction between GI and GII.

	GI (n=38)	GII (n=30)	*p*-value
**Stress LVEF (%)**	43 (26 - 60)	74.5 (70 - 80.75)	< 0.001
**Rest LVEF (%)**	39.5 (21 - 55)	68.5 (60 - 74)	< 0.001
**Stress ESV (ml)**	84 (50 - 130)	24 (21 - 32)	0.07
**Rest ESV (ml)**	78.5 (37.25 - 129.25)	30 (21.25 - 39.25)	< 0.001
**Stress EDV (ml)**	147 (98 - 185)	91 (82.25 - 105)	< 0.001
**Rest EDV (ml)**	136 (80 - 176.75)	86.5 (73 - 97.5)	0.001

LVEF = left ventricular ejection fraction; ESV= end systolic volume; EDV = end diastolic volume.

**Table 5:** Results of the comparisons of left ventricular ejection fraction between GI-A, GI-B, and GII.

	GI-A (n=12)	GI-B (n=26)	*p-value♦*	GII (n=30)	p-value*
**Stress LVEF (%)**	35 (24 - 42.5)	51.5 (26.5 - 61.5)	0.18	74.5 (70 - 80.75)	< 0.01
**Rest LVEF (%)**	30.5 (22.75 - 41.75)	43 (21 - 56.75)	0.50	68.5 (60 - 74)	< 0.01
**Stress ESV (ml)**	109 (72.5 - 166)	74.5 (47.5 - 121.5)	0.32	24 (21 - 32)	< 0.01
**Rest ESV (ml)**	98 (70.25 - 151.5)	69 (26 - 124.75)	0.25	30 (21.25 - 39.25)	< 0.01
**Stress EDV (ml)**	171 (126 - 214.5)	137.5 (96.5 - 173.75)	0.26	91 (82.25 - 105)	0.03
**Rest EDV (ml)**	153 (96.75 - 193.25)	119 (58 - 160)	0.25	86.5 (73 - 97.5)	0.24

♦ refers to the comparison between GI-A *vs*. GI-B; * refers to the comparison between GII *vs*. GI-B.

LBBB = left bundle branch block; LVEF = left ventricular ejection fraction; ESV= end systolic volume; EDV = end diastolic volume.

## References

[1] Vaduganathan P, He ZX, Raghavan C, Mahmarian JJ, Verani MS (1996). Detection of left anterior descending coronary artery stenosis in patients with left bundle branch block: exercise, adenosine or dobutamine imaging. J Am Coll Cardiol.

[2] Nallamothu N, Bagheri B, Acio ER, Heo J, Iskandrian AE (1997). Prognostic value of stress myocardial perfusion single photon emission computed tomography imaging in patients with left bundle branch block. J Nucl Cardiol.

[3] Hirzel HO, Senn M, Nuesch K, Buettner C, Peiffer A, Hess OM (1984). Thallium-201 scintigraphy in complete left bundle branch block. Am J Cardiol.

[4] Huerta EM, Padial LR, Beiras JMC, Illera JP, Cardiel EA (1987). Thallium-201 exercise scintigraphy in patients having complete left bundle branch block with normal coronary arteries. Int J Cardiol.

[5] DePuey EG, Guertler-Krawczynska E, Robbins WL (1988). Thallium-201 SPECT in coronary artery disease patients with left bundle branch block. J Nucl Med.

[6] Larcos G, Gibbons RJ, Brown ML (1991). Diagnostic accuracy of exercise thallium-201 single photon emission computed tomography in patients with left bundle branch block. Am J Cardiol.

[7] Matzer L, Kiat H, Friedman JD, Van Train K, Maddahi J, Berman DS (1991). A new approach to the assessment of tomography thallium-201 scintigraphy in patients with left bundle branch block. J Am Coll Cardiol.

[8] Tawarahara K, Kurata C, Taguchi T, Kobayashi A, Yamazaki N (1992). Exercise testing and tallium-201 emission computed tomography in patients with intraventricular conduction disturbances. Am J Cardiol.

[9] Kay J, Dorbala S, Goyal A, Fazel R, Di Carli MF, Einstein AJ (2013). Influence of sex on risk stratification with stress myocardial perfusion Rb-82 positron emission tomography. Results from the PET (Positron Emission Tomography) prognosis multicenter study. J Am Coll Cardiol.

[10] Ghotbi AA, Kjaer A, Hasbak P (2014). Review: comparison of PET rubidium-82 with conventional SPECT myocardial perfusion imaging. Clin Physiol Funct Imaging.

[11] Skalidis EI, Kochiadakis GE, Koukouraki SI, Parthenakis FI, Karkavitsas NS, Vardas P (1999). Phasic coronary flow pattern and flow reserve in patients with left bundle branch block and normal coronary arteries. J Am Coll Cardiol.

[12] Gould KL, Johnson NP, Bateman TM, Beanlands RS, Bengel FM, Bober R (2013). Anatomic versus physiologic assessment of coronary artery disease. Role of coronary flow reserve, fractional flow reserve, and positron emission tomography imaging in revascularization decision-making. J Am Coll Cardiol.

[13] Sdringola S, Johnson N, Kirkeeide RL, Cid E, Gould L (2011). Impact of unexpected factors on quantitative myocardial perfusion and coronary flow reserve in young, asymptomatic volunteers. JACC Cardiovasc Imaging.

[14] Hoffman JI (2000). Problems of coronary flow reserve. Ann Biomed Eng.

[15] Dorbala S, Di Carli MF, Delbeke D, Abbara S, DePuey G, Dilsizian V (2013). SNMMI/ASNC/SCCT Guidelines for cardiac SPECT/CT and PET/CT. J Nucl Med.

[16] Senthamizhchelvan S, Bravo PE, Lodge MA, Merrill J, Bengel FM, Sgouros G (2011). Radiation dosimetry of <sup>82</sup>Rb in humans under pharmacologic stress. J Nucl Med.

[17] Germano G, Kiat H, Kavanagh PB, Moriel M, Mazzanti M, Su H (1995). Automatic quantification of ejection fraction from gated myocardial perfusion SPECT. J Nucl Med.

[18] DeKemp RA, Declerck J, klein R, Pan XB, Nakazato R, Tonge C (2013). Multisoftware reproducibility study of stress and rest myocardial blood flow assessed with 3-D dynamic PET/CT and 1-tissue-compartment model of 82Rb kinetics. J Nucl Med.

[19] Lortie M, Beanlands RS, Yoshinaga K, Klein R, Dasilva JN, deKemp RA (2007). Quantification of myocardial blood flow with 82Rb dynamic PET imaging. Eur J Nucl Med Mol Imaging.

[20] Klocke FJ, Baird MG, Lorell BH, Bateman TM, Messer JV, Berman DS (2003). ACC/AHA/ASNC Guidelines for the clinical use of cardiac radionuclide imaging. A report of the American College of Cardiology/ American Heart Association task force on practice guidelines (ACC/AHA/ASNC committee to revise the 1995 guidelines for the clinical use of cardiac radionuclide imaging). www.acc.org.

[21] Munzel U, Hothorn LA (2001). A unified approach to simultaneous rank tests procedures in the unbalanced one-way layout. Biom J.

[22] Konietschke F, Bathke AC, Hothorn LA, Brunner E (2010). Testing and estimation of purely nonparametric effects in repeated measures designs. Computational Statistics & Data Analysis.

[23] R Core Team R: A language and environment for statistical computing. 2014, R Foundation for Statistical Computing, Vienna, Austria. http://www.R-project.org/.

[24] Ziadi MC, dekemp RA, Williams KA, Guo A, Chow BJW, Renaud JM (2011). Impaired myocardial flow reserve on rubidium-82 positron emission tomography imaging predicts adverse outcomes in patients assessed for myocardial ischemia. J Am Coll Cardiol.

[25] Taqueti VR, Hachamovitch R, Murthy VL, Naya M, Foster CR, Hainer J (2015). Global coronary flow reserve is associated with adverse cardiovascular events independently of luminal angiographic severity and modifies the effect of early revascularization. Circulation.

[26] Fahy GJ, Pinski SL, Miller DP, McCabe N, Pye C, Walsh MJ (1996). Natural history of isolated bundle branch block. Am J Cardiol.

[27] Schneider JF, Thomas Jr HE, Sorlie P, Kreger BE, McNamara PM, Kannel WB (1981). Comparative features of newly acquired left and right bundle branch block in the general population: The Framingham study. Am J Cardiol.

[28] Jukema JW, Van Der Wall EE, Van Der Vis-Melsen MJE, Kruyswijk HH, Bruschke AVG (1993). Dipyridamole thallium-201 scintigraphy for improved detection of left anterior coronary artery stenosis in patients with left bundle branch block. Eur Heart J.

[29] Grines CL, Bashore TM, Boudoulas H, Olson S, Shafer P, Wooley CF (1989). Functional abnormalities in isolated left bundle branch block: the effect of interventricular asynchrony. Circulation.

[30] Burns RJ, Galligan L, Wright LM, Lawand S, Burke RJ, Gladstone PJ (1991). Improved specificity of myocardial thallium 201 single photon emission computed tomography in patients with left bundle branch block by dipyridamole. Am J Cardiol.

[31] Ebersole MDG, Heironimus LCJ, Toney LCMO, Billingsley CJ (1993). Comparison of exercise and adenosine technetium-99m sestamibi myocardial scintigraphy for diagnosis of coronary artery disease in patients with left bundle branch block. Am J Cardiol.

[32] O'Keefe Jr JH, Bateman TM, Barnhart CS (1993). Adenosine thallium-201 is superior to exercise thallium-201 for detecting coronary artery disease in patients with left bundle branch block. J Am Coll Cardiol.

[33] Masci PG, Marinelli M, Piacenti M, Lorenzoni V, Positano V, Lombardi M (2010). Myocardial structural, perfusion, and metabolic correlates of left bundle branch block mechanical derangement in patients with dilated cardiomyopathy: a tagged cardiac magnetic resonance and positron emission tomography study. Cir Cardiovasc Imaging.

[34] Koepfli P, Wyss CA, Gaemperli O, Siegrist PT, Klainguti M, Schepis T (2009). Left bundle branch block causes relative but not absolute septal underperfusion during exercise. Eur Heart J.

[35] Rowe DW, DePuey EG, Sonnemaker RE, Hall RJ, Burdine JA (1983). Left ventricular performance during exercise in patients with left bundle branch block: evaluation by radionuclide ventriculography. Am Heart J.

[36] Herrmann J, Kaski JC, Lerman A (2012). Coronary microvascular dysfunction in the clinical setting: from mystery to reality. Eur Heart J.

[37] Tsagalou EP, Anastasiou-Nana M, Agapitos E, Gika A, Drakos SG, Terrovitis JV (2008). Depressed coronary flow reserve is associated with decreased myocardial capillary density in patients with heart failure due to idiopathic dilated cardiomyopathy. J Am Coll Cardiol.

[38] Majmudar MD, Murth VL, Shah RV, Kolli S, Mousavi N, Foster CR (2015). Quantification of coronary flow reserve in patients with ischaemic and non-ischaemic cardiomyopathy and its association with clinical outcomes. Eur Heart J Cardiovasc Imaging. Eur Heart J Cardiovasc Imaging.

[39] Jaarsma C, Leiner T, Bekkers SC, Crijns HJ, Wildberger JE, Nagel E (2012). Diagnostic performance of noninvasive myocardial perfusion imaging using single-photon emission computer tomography, cardiac magnetic resonance, and positron emission tomography imaging for the detection of obstruction coronary artery disease. J Am Coll Cardiol.

